# Identification of environmentally stable QTL for resistance against *Leptosphaeria maculans* in oilseed rape (*Brassica napus*)

**DOI:** 10.1007/s00122-015-2620-z

**Published:** 2015-10-30

**Authors:** Y. J. Huang, C. Jestin, S. J. Welham, G. J. King, M. J. Manzanares-Dauleux, B. D. L. Fitt, R. Delourme

**Affiliations:** Centre for Agriculture, Food and Environmental Management, University of Hertfordshire, Hatfield, Hertfordshire, AL10 9AB UK; Rothamsted Research, Harpenden, Hertfordshire, AL5 2JQ UK; Terres Inovia, 78850 Thiverval-Grignon, France; VSN International Ltd, Waterhouse Street, Hemel Hempstead, Hertfordshire, HP1 1ES UK; Southern Cross University, Lismore, NSW 2480 Australia; INRA, UMR1349 IGEPP, BP 35327, 35653 Le Rheu Cedex, France; Agrocampus Ouest, UMR1349 IGEPP, BP 35327, 35653 Le Rheu Cedex, France

## Abstract

**Key message:**

**Six stable QTL for resistance against*L. maculans*(phoma stem canker) have been identified by QTL × environment interaction analysis using data from five winter oilseed rape field experiments**.

**Abstract:**

Phoma stem canker, caused by *Leptosphaeria maculans*, is a disease of worldwide importance on oilseed rape (*Brassica napus*). Quantitative trait loci (QTL)-mediated resistance against *L. maculans* in *B. napus* is considered to be race non-specific and potentially durable. Identification and evaluation of QTL for resistance to *L. maculans* is important for breeding oilseed rape cultivars with durable resistance. An oilseed rape mapping population was used to detect QTL for resistance against *L. maculans* in five winter oilseed rape field experiments under different environments. A total of 17 QTL involved in ‘field’ quantitative resistance against *L. maculans* were detected and collectively explained 51 % of the phenotypic variation. The number of QTL detected in each experiment ranged from two to nine and individual QTL explained 2–25 % of the phenotypic variation. QTL × environment interaction analysis suggested that six of these QTL were less sensitive to environmental factors, so they were considered to be stable QTL. Markers linked to these stable QTL will be valuable for selection to breed for effective resistance against *L. maculans* in different environments, which will contribute to sustainable management of the disease.

**Electronic supplementary material:**

The online version of this article (doi:10.1007/s00122-015-2620-z) contains supplementary material, which is available to authorized users.

## Introduction

The use of quantitative resistance (mediated by Quantitative Trait Loci, QTL) against pathogens of arable crops that cause devastating epidemics makes an essential contribution to global food security in a changing climate (Brun et al. 2010; Flood 2010; Fitt et al. 2011; Mahmuti et al. 2009; St Clair 2010; Oerke 2006; Stern 2007). Generally, quantitative resistance is considered to be more durable and less likely to be rendered ineffective by new virulent pathogen races than resistance mediated by single *R* genes that is frequently associated with ‘boom and bust’ cycles (Delourme et al. 2014; Brun et al. 2010; Stukenbrock and McDonald 2008; Poland et al. 2008; St Clair 2010). The use of such quantitative resistance is vital for non-intensive cropping systems where farmers cannot afford fungicides and their crops are threatened by widespread epidemics of diseases (Schmidhuber and Tubiello 2007; Flood 2010).

Phoma stem canker (also called blackleg) is a disease of brassica crops with worldwide importance that causes serious epidemics in Australia, America and Europe (Howlett, 2004; Fitt et al. 2006a); for example, it was estimated to cause worldwide losses valued at >£1000 M per cropping season in oilseed rape (*Brassica napus*) at a price of £370/t (Fitt et al. 2011). Serious epidemics are associated with the stem canker pathogen *Leptosphaeria maculans* (anamorph *Plenodomus lingam*), which has been spreading globally over the last 30 years and now threatens oilseed rape production in China, where currently only the less damaging *L. biglobosa* (anamorph *Plenodomus biglobosus*) occurs (Fitt et al. 2006b, 2008; Zhang et al. 2014; Liu et al. 2014). Furthermore, the severity of epidemics is predicted to increase with global warming (Evans et al. 2008; Butterworth et al. 2010). The use of host resistance to control this disease is becoming ever more important. Resistance against *L. maculans* relies on two types of resistance that operate in *B. napus* (Delourme et al. 2006; Rimmer 2006).

Firstly, major resistance (*R*) gene-mediated resistance operates at the leaf infection stage, after air-borne ascospores produced on crop debris have landed on leaves of the new crop, germinated and begun to penetrate leaves through stomata (West et al. 2001; Huang et al. 2003). This occurs in autumn in northern Europe, including the UK, where oilseed rape crops are generally winter (autumn-sown) types. *R* gene-mediated resistance protects the plant from development of phoma leaf spots at the young plant stage (i.e. no phoma leaf spots in autumn) and subsequently prevents the development of phoma stem canker at the adult plant stage (e.g. no phoma stem canker in the following spring/summer). Therefore, *R* gene-mediated resistance against *L. maculans* is also referred as complete resistance. Secondly, quantitative resistance, mediated by QTL, operates as the pathogen is spreading symptomlessly along the leaf petiole towards the stem or growing in stem tissues (Huang et al. 2009, 2014). Quantitative resistance does not prevent the development of phoma leaf spots at the young plant stage but decreases the severity of phoma stem canker at the adult plant stage. Therefore, quantitative resistance against *L. maculans* is also referred as partial resistance (Delourme et al. 2006). Successful breeding of oilseed rape cultivars for control of phoma stem canker in Australia and France has led to an improvement in quantitative resistance with time (Cowling 2007; Jestin et al. 2011). Since *R* gene-mediated resistance is race-specific and is often rapidly rendered ineffective by changes in *L. maculans* populations if it is deployed commercially in a large area over more than three years (Rouxel et al. 2003; Sprague et al. 2006), combining *R* gene resistance with quantitative resistance provides a more robust crop protection strategy (Brun et al. 2010; Delourme et al. 2014). However, effective detection of quantitative resistance in field conditions is only possible in the absence of effective *R* genes. Therefore, QTL for resistance against *L. maculans* can be identified only in mapping populations or in germplasm collections that do not segregate for effective *R* genes.

Few previous studies have identified QTL for resistance against *L. maculans*. One French winter oilseed rape cultivar Darmor was used as a source of resistance in two genetic backgrounds (Pilet et al. 1998, 2001; Jestin et al. 2012). Pilet et al. (2001) showed that both the genetic background and the environment influenced detection of QTL. However, four QTL for resistance to *L. maculans* were detected consistently. QTL analyses done in Australia in different populations also showed that environmental conditions influenced detection of resistance QTL (Kaur et al. 2009; Raman et al. 2012). One of the limitations in the use of resistance QTL in breeding is their inconsistency due to genotype × environment interactions (Poland et al. 2008; McDonald 2010). It is essential for breeders to develop oilseed rape cultivars with resistance that is effective in different environmental conditions. Although four QTL for resistance to *L. maculans* were consistently detected in two genetic backgrounds over two growing seasons in France (Pilet et al. 1998, 2001), it was not clear whether these QTL could be considered as stable QTL. There is evidence that environmental factors, especially temperature, affect the effectiveness of both *R* gene-mediated resistance and quantitative resistance against *L. maculans* (Huang et al. 2006, 2009). Identification of stable resistance is important both for breeding and for understanding mechanisms of resistance. This paper describes identification of *B. napus* QTL for resistance against *L. maculans* that are less sensitive to environmental factors, thus facilitating selection of stable QTL in breeding for effective, stable resistance.

## Materials and methods

### Mapping population

The segregating doubled haploid (DH) mapping population BnaDYDH, derived from the cross Darmor-*bzh* × Yudal, was developed in France (Foisset et al. 1996). The parent Darmor-*bzh*, with good quantitative resistance against *L. maculans*, is a dwarf line that is near isogenic to the French winter oilseed rape cultivar Darmor. Yudal is a Korean spring oilseed rape line that is very susceptible to *L. maculans*. The BnaDYDH population does not segregate for *R* genes that are effective in the UK or France. Different sets of DH lines from the BnaDYDH population were evaluated in France and the UK in five winter oilseed rape field experiments over different cropping seasons (Table 1).

**Table 1:** Numbers of DH (doubled haploid) lines from the BnaDYDH (Darmor-*bzh* × Yudal) oilseed rape mapping population grown, phoma stem canker severity (G2 index) assessed before harvest and heritability of resistance against *Leptosphaeria maculans* in five winter oilseed rape field experiments

Experiment	Site	Harvest year	No. of DH lines	Stem canker severity (range)^a^	Heritability
INRA95	Le Rheu, France	1995	171	6.03 (2.64–8.14)	0.84
INRA96	Le Rheu, France	1996	171	4.43 (1.18–6.33)	0.82
INRA07	Le Rheu, France	2007	279	5.13 (1.84–7.76)	0.94
RRes08	Rothamsted, UK	2008	120	1.97 (0.46–4.99)	0.82
RRes09	Rothamsted, UK	2009	120	3.11 (1.03–7.00)	0.89

^a^Phoma stem canker severity was assessed on 25 or 40 plants from each plot on a 1–6 scale, and then data were used to calculate a G2 index; data presented are the mean and range of the G2 index for all the DH lines in each experiment

### Field experiments

Field experiments with 171 DH lines from the BnaDYDH mapping population were done in the 1994/1995 (INRA95) and 1995/1996 (INRA96) cropping seasons in randomised incomplete block designs with three replicates at Le Rheu, INRA, France (Pilet et al. 1998). In 2006/2007 (INRA07), a field experiment with 279 DH lines in a randomised incomplete block design with three replicates was done at Le Rheu, INRA, France (Jestin et al. 2012). Each replicate included a 5-row plot (2.5 m^2^) of each DH line and the parental lines (Darmor-*bzh*, Yudal). In France, five commercial winter oilseed rape cultivars (Aviso, Darmor, Eurol, Falcon and Jet Neuf) that differed in quantitative resistance against *L. maculans* were included as controls (S-Table 1). In 2007/2008 and 2008/2009, field experiments were done with 120 DH lines in a randomised incomplete block design with three replicates at Rothamsted Research (RRes), UK. Each replicate included a 10-row plot (6 m^2^) of each DH line and the parental lines. In the UK, five commercial winter oilseed rape cultivars (Aviso, Canberra, Darmor, Eurol and NK-Bravour) that differed in quantitative resistance were also included as controls (S-Table 1). In each cropping season, oilseed rape stubble affected by *L. maculans* that had been collected after harvest at the end of the previous season was scattered across the field experiment at a density of one stem (at Rothamsted, UK) or two stems (at INRA, France) per m^2^ when the crop was at the two to three leaves growth stage.

Stem canker severity was assessed in late June on a 1–6 scale when plants were just starting to senesce, and the data were then converted to a G2 disease index (Pilet et al. 1998; Delourme et al. 2008a). Forty (1995, 1996, 2007) or 25 (2008, 2009) plants were uprooted from each plot and phoma stem canker was scored on a 1–6 scale, based on the internal area of necrosis at the stem-base cross section: 1 = no affected tissue; 2 = 1–5 % area affected; 3 = 6–50 % area affected; 4 = 51–75 % area affected; 5 = 76–100 % area affected, plant alive, and 6 = 100 % area affected, stem broken or plant dead. The stem canker severity data were then used to calculate the G2 index by the formula: G2 index = [(*N*1 × 0) + (*N*2 × 1) + (*N*3 × 3) + (*N*4 × 5) + (*N*5 × 7) + (*N*6 × 9)]/*N*t, where *N*1, 2…6 are the numbers of stems with canker scores 1, 2…6, respectively, and *N*t is the total number of stems assessed.

In each season, the weather data (e.g. rainfall and temperature) were collected from on-site weather stations; the accumulated temperature (degree-days above 0 °C) from the date in autumn when 10 % of plants were observed with phoma leaf spots to the date before harvest in the following summer when phoma stem canker was assessed was calculated for each growing season.

### Statistical analysis and QTL mapping

All the analyses were done using GENSTAT statistical software (Payne et al. 2011). Data from the field experiments were analysed as mixed models. Preliminary analysis indicated that transformation was required to normalise the residual variance for all traits; logit transformation [log(G2_index/(9–G2_index))] was used on data from all experiments for the stem canker severity index. The presence of a spatial trend was investigated for Rothamsted experiments, where the experimental spatial layout was available. The best spatial model was compared to a design-based model (incomplete block design) and the model with the smallest value of the Bayesian Information Criterion (BIC) (Verbeke and Molenberghs 2000) was selected. The design-based model was used for all traits assessed in INRA experiments. Two restricted maximum likelihood (REML) analyses were done for each trait within each experiment: (a) with DH line effects fitted as random effects, to enable calculation of heritability, and (b) with DH line effects fitted as fixed effects, to provide predicted means for use in QTL analysis. The Pearson correlation coefficient was calculated using the predicted line means (logit scale) for the five experiments. Heritability was quantified in the random lines model using a generalised heritability coefficient (Cullis et al. 2006), which provides a mean line value calculated as

$$1 - \frac{{{\text{Mean}}({\text{p}} . {\text{e}} . {\text{v}} .)}}{{\sigma_{g}^{2} }},$$

where *σ*_g_^2^ is the estimated genetic variance and p.e.v. is the prediction error variance of the estimated line effects.

Based on published maps for the BnDYDH population (Delourme et al. 2008b; Wang et al. 2011a) and new markers that have been recently developed (Jestin et al. 2012), a set of 505 markers was chosen to construct a new map (S-Table 2). This map was used for QTL detection. QTL analysis was based on genetic predictors calculated at intervals within the genetic map. Where pairs of markers were coincident, the marker with the fewest genotype scores was omitted. Simple interval mapping was used to identify individual QTL, and then composite interval mapping was used to construct a model for combined QTL effects. Simple interval mapping and composite interval mapping were implemented by regression on genetic predictors at specified intervals (Haley and Knott 1992). The trait data for this analysis were the predicted line means from the individual experiments. Genetic predictors were calculated at intervals of 5 cM. Thresholds corresponding to genome-wide significance levels were calculated using the method of Li and Ji (2005). QTL analyses were first done separately for each experiment. The model identification procedure started with simple interval mapping using a genome-wide significance level of 0.1. Candidate QTL were used as initial cofactors for composite interval mapping, followed by two rounds each of forward and backward selection. All candidate QTL identified in this process were then used in regression with all subsets, and the subset with the largest adjusted *R*^2^ statistic, subject to all effects exceeding the 0.05 genome-wide significance threshold, was selected. QTL effects were estimated for this final model, and the percentage variance accounted for was calculated as a relative decrease in the residual variance. The support interval was defined as the interval where the statistics (−log*P*) decreased by one unit on both sides of the maximum.

For a combined analysis across sites, a mixed model was used, with both genotype and QTL × experiment effects as random effects and experiments as fixed effects. Effects of QTL × environment interactions were estimated using the mixed model approach (Malosetti et al. 2004, Boer et al. 2007; van Eeuwijk et al. 2010). REML estimation was used with a simple model selection procedure. An unstructured covariance matrix was used to model covariance patterns across sites/years and to allow for heterogeneity in line variances across sites/years. Simple interval mapping in terms of combined QTL main effects and QTL × experiment interactions provided candidate QTL locations used in composite interval mapping, with two rounds of alternate forward and backward selection, leading to the final model. A final round of backward selection was used to exclude non-significant QTL × experiment interactions from this model, enabling identification of QTL locations with a consistent effect across experiments. The total genetic variance in the data was quantified as the trace of the covariance matrix for genotype × experiment interactions in the absence of QTL effects. The percentage of the variation accounted for by QTL models was quantified as the change in this trace term each time that a QTL or QTL × experiment term was added into the model. Primer information for the marker loci located at or near the QTL peak is given in S-Table 3.

## Results

### Phenotypic analysis

**Fig. 1:**
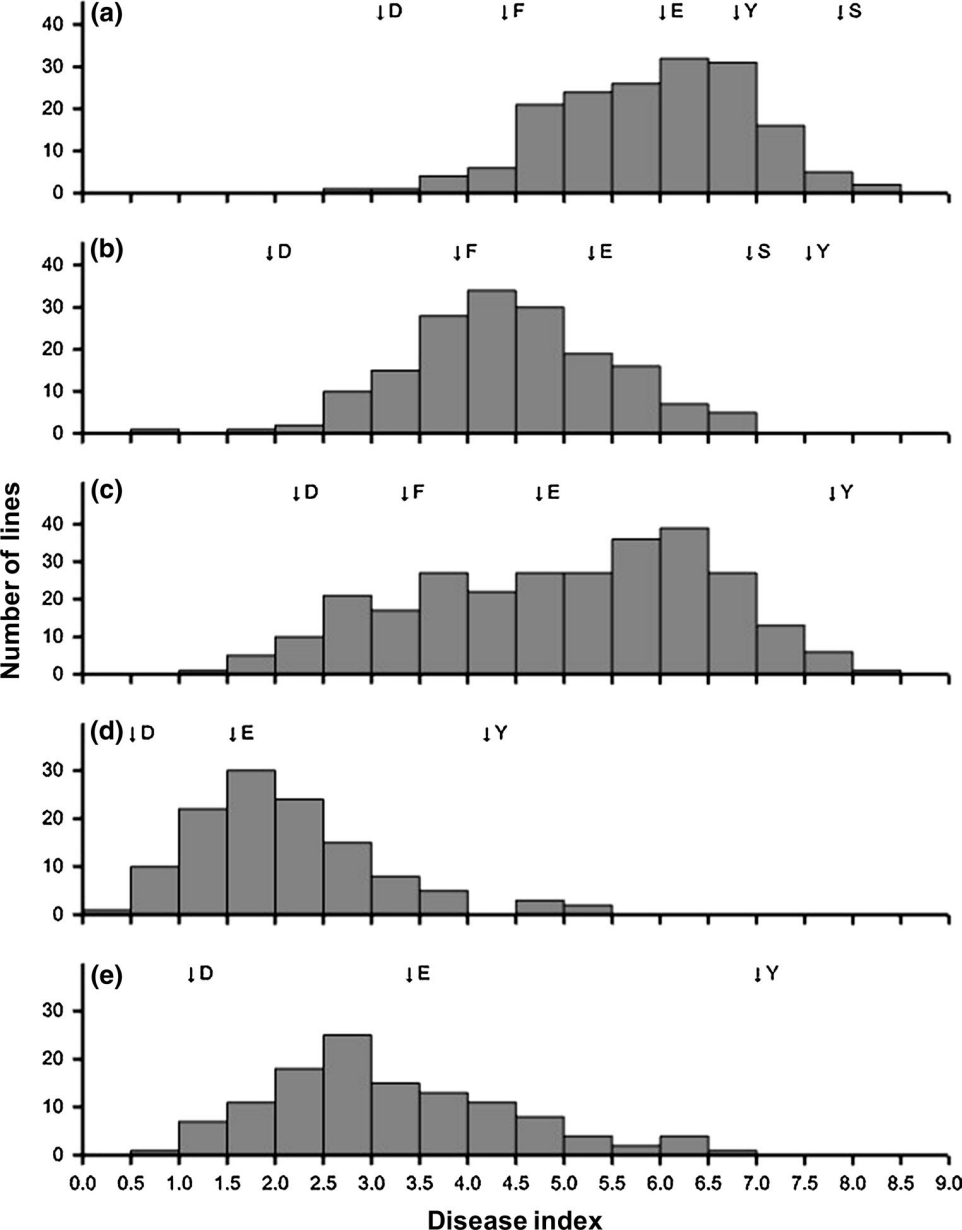
Frequency (number of DH lines) distributions of phoma stem canker severity (G2 index) for lines in the BnaDYDH (Darmor-*bzh* × Yudal) mapping population in five winter oilseed rape field experiments: **a** INRA-Rennes, France, harvest year 1995; **b** INRA-Rennes, France, harvest year 1996; **c** INRA-Rennes, France, harvest year 2007; **d** Rothamsted, UK, harvest year 2008; **e** Rothamsted, UK, harvest year 2009). Arrows indicate the disease index for the mapping population parents (*D* Darmor-*bzh*, *Y* Yudal) and some of the elite cultivars (*E* Eurol, *F* Falcon, *S* Shogun) included as controls

The G2 disease index frequency distribution patterns differed between the five experiments (Fig. 1), but the continuous unimodal shape of the distributions was consistent. This confirmed that the resistance against *L. maculans* in the BnaDYDH population was quantitative. A large range in phoma stem canker severity (expressed as G2 index) was observed within each experiment and the mean G2 disease index was greater in France than in the UK (Table 1). For example, the G2 index of Eurol, used as a control in all five experiments, was smaller in the UK (mean 2.7) than in France (mean 5.3) (Fig. 1; S-Table 1). The heritability of quantitative resistance was high within each experiment and similar in England and France over the 5 years (Table 1). The correlation coefficients for line predictions across the five experiments were positive and significant (*P* < 0.001) (Table 2), indicating some consistency in line ranking across the experiments. The G2 index of the resistant parent Darmor-*bzh* was similar to those of resistant control cultivars in the five experiments (Fig. 1), suggesting that Darmor remains a good source of quantitative resistance against *L. maculans*.

**Table 2:** Correlation coefficient values for relationships between the different phoma stem canker severity scores (logit-transformed G2 index) assessed before harvest in each of five winter oilseed rape field experiments with DH lines from the BnaDYDH (Darmor-*bzh* × Yudal) mapping population

Experiment^a^	Correlation coefficient				
	INRA95	INRA96	INRA07	RRes08	RRes09
INRA95	1.00^b^				
INRA96	0.54	1.00			
INRA07	0.71	0.52	1.00		
RRes08	0.44	0.49	0.64	1.00	
RRes09	0.53	0.63	0.64	0.63	1.00

^a^Number of DH lines and other details of each experiment given in Table 1

^b^All relationships, *P* < 0.001

### Identification of QTL for resistance against *L. maculans*

**Fig. 2:**
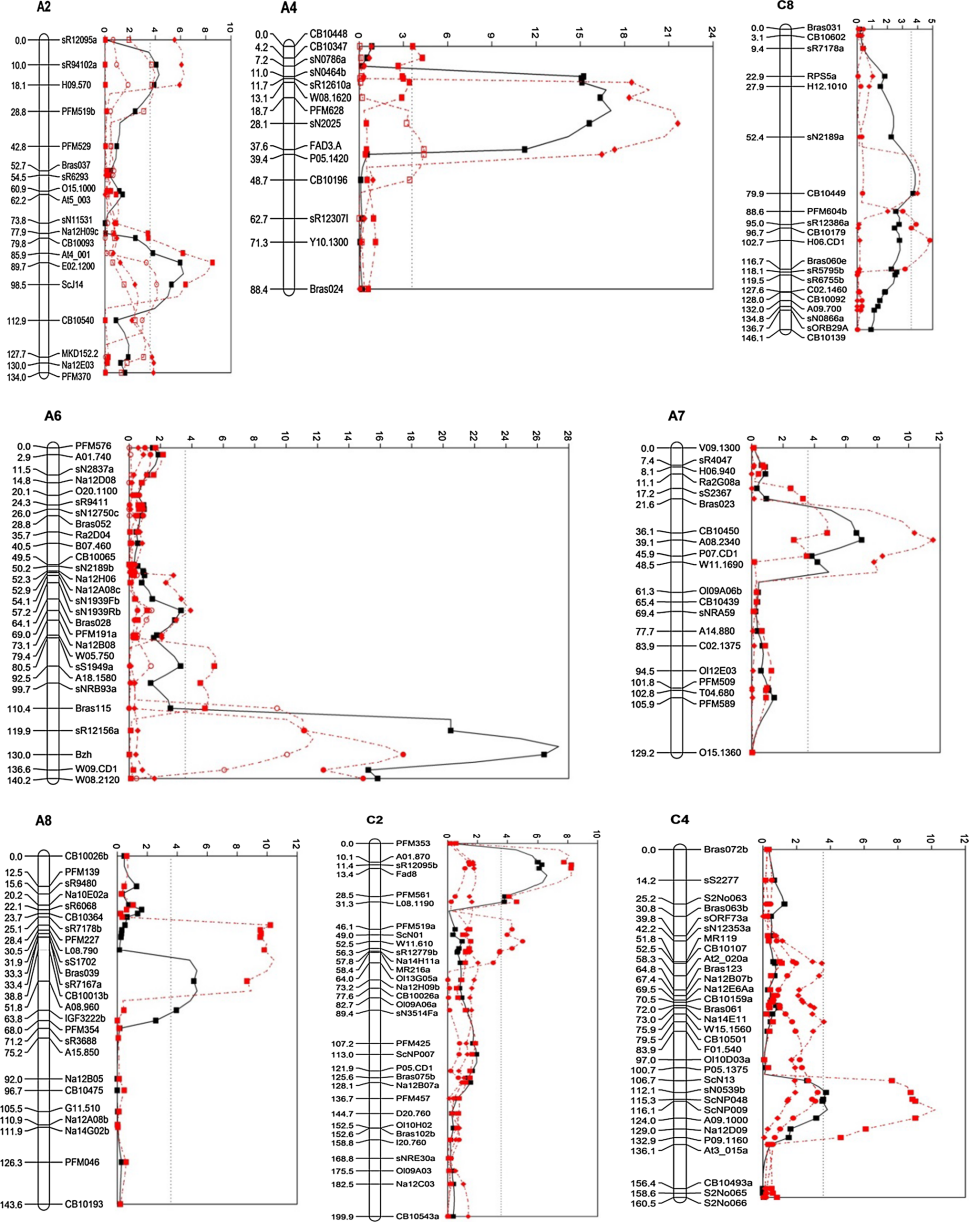
Test statistic (-log*P*) for QTL effects (or QTL × experiment effects for the combined years) at steps of 5 cM in the composite interval mapping model for resistance against *Leptosphaeria maculans* in the BnaDYDH (Darmor-*bzh* × Yudal doubled haploid) mapping population in five winter oilseed rape field experiments: INRA95 (INRA-Rennes, France 1994/1995), INRA96 (INRA-Rennes, France 1995/1996), INRA07 (INRA-Rennes, France 2006/2007), RRES08 (Rothamsted, UK, 2007/2008) and RRES09 (Rothamsted, UK, 2008/2009). The linkage groups are named according to *B. napus* A1-A10 and C1-C9 designations. Data analysed for QTL effects were logit(G2 index) combined across the five experiments (*black square*): logit(G2 index) in INRA95 (*red filled square*); logit(G2 index) in INRA96 (*red filled circle*); logit(G2 index) in INRA07 (*red filled diamond*); logit(G2 index) in RRES08 (*red square*) and logit(G2 index) in RRES09 (*red circle*)

The QTL for resistance against *L. maculans* detected in the individual analyses of each of the five experiments were located in 17 genomic regions distributed over 13 linkage groups (Table 3). The estimated phenotypic variation explained by individual QTL ranged from 2 to 25 %, with overall phenotypic variation explained ranging from 30 to 55 %. In this new analysis, eight QTL were detected in INRA95, four in INRA96 and nine in INRA07, while seven had been detected previously in INRA95, five in INRA96 (Pilet et al. 1998) and ten in INRA07 (Jestin et al. 2012) (S-Table 4). In the new analysis, a new QTL was detected on A4 in the INRA95 experiment. Two QTL previously detected on A1 and C4 in the INRA96 experiment were not detected in the new analysis, but a new QTL was detected on C7. One QTL on linkage group A6 detected both in France (INRA96) and in the UK (RRes09) was linked to the dwarf gene (*bzh*) on A6. Another QTL was also detected on A6 in France (INRA95 and INRA07) but at some distance from the dwarf gene (Fig. 2). In the INRA07 experiment, nine QTL were detected, with individual QTL explaining 2–15 % of the variance; collectively, these QTL explained 45 % of the variance. Compared to the two other cropping seasons in France and the two seasons in the UK, two new QTL were detected in the INRA07 experiment; one QTL on A1 had large effect and the other one on A3 had small effect.

**Table 3:** Information about QTL for resistance against *Leptosphaeria maculans* detected by composite interval mapping in each of five winter oilseed rape field experiments with the BnaDYDH (Darmor-*bzh* × Yudal) mapping population

LG^a^	Locus^b^	Position (cM)^b^	Support interval (cM)	−log (*P*)^c^	Effect (SE)^d^	*R* ^2^ (%)^e^
INRA95						
A2	E02.1200	89.7	85.9–98.5	8.54	0.18 (0.03)	6.64
A4	CB10347	4.2	0.0–7.2	4.28	0.12 (0.03)	3.01
A6	A18.1580	88.5	80.5–110.4	5.51	0.15 (0.03)	4.55
A7	CB10450	31.3	21.6–39.1	4.82	0.14 (0.03)	2.96
A8	CB10013b	43.1	28.4–55.8	10.46	0.21 (0.03)	7.47
A9	W15.1470	124.8	110.3–129.4	6.88	0.18 (0.03)	7.12
C2	Fad8	17.2	3.4–24.7	8.25	−0.18 (0.03)	7.55
C4	A09.1000	120.0	115.3–124.0	10.22	0.20 (0.03)	5.63
INRA96						
A6	Bzh	130.0	126.6–133.2	17.47	0.27 (0.03)	24.33
C2	W11.610	52.5	41.2–57.8	5.02	−0.14 (0.03)	2.59
C7	Na12A10	123.1	111.0–137.3	5.25	0.15 (0.03)	3.63
C8	H06.CD1	102.7	95.0–116.7	4.81	0.13 (0.03)	7.59
INRA07						
A1	sN2305a	104.9	102.5–112.0	7.93	0.18 (0.03)	7.69
A2	sR94102a	5.0	0–21.6	6.18	0.21 (0.04)	4.23
A3	Na12C07	215.6	200.9–221.3	3.68	−0.13 (0.03)	1.68
A4	sN2025	28.1	18.7–32.9	21.61	0.35 (0.04)	15.17
A6	PFM191a	69.0	57.2–76.2	3.91	0.13 (0.03)	1.82
A7	A08.2340	39.1	36.1–42.5	11.55	0.23 (0.03)	5.24
A9	ScL12	105.9	104.5–117.2	6.06	0.17 (0.03)	5.24
C7	Bras014	83.5	68.6–92.8	5.98	0.16 (0.03)	1.51
C8	CB10449	70.7	61.0–84.2	4.14	0.15 (0.04)	3.20
RRes08						
A2	sR94102a	14.1	5.0–28.8	4.00	0.19 (0.05)	9.16
A4	FAD3.A	37.6	23.4–48.7	4.39	0.19 (0.05)	12.88
C1	sN11707a	21.2	3.5–26.8	4.29	−0.19 (0.05)	11.16
RRes09						
A2	ScJ14	103.3	89.7–117.8	4.16	0.21 (0.05)	5.68
A6	sR12156a	123.3	110.4–133.3	11.73	0.34 (0.05)	24.80

^a^LG, the linkage groups, are named according to *Brassica napus* A1–A10 and C1–C9 designations by the Multinational *Brassica* Genome Project Steering Committee

(http://www.brassica.info/information/lg_assigments.htm)

^b^The marker closest to the position of the maximum effect of the QTL

^c^Test statistic value for QTL

^d^The additive effect (standard error given in brackets)

^e^Proportion (%) of the phenotypic variation explained by the QTL

In the RRes08 experiment, three QTL for resistance associated with stem canker severity assessed in summer were detected, with individual QTL accounting for 9–13 % of the variance. The QTL on C1 was not detected in the other four experiments. In the RRes09 experiment, two QTL detected, on A2 and A6 (Table 3), individually explained 6 and 25 % of the variance, respectively. These two QTL were located in the same region as two QTL detected in 1994/1995 and 1995/1996 field experiments in France but at different locations (Fig. 2).

### Effects of environmental factors on detection of QTL for resistance against *L. maculans*

When the logit-transformed G2 disease index data from the five experiments were analysed together, nine QTL were detected (Fig. 2; Table 4). The percentages of the variance accounted for by the QTL main effects (that were consistent across experiments) and by genotype × environment interactions differed between the QTL. The variance accounted for by the main effects of individual QTL varied from 2 to 7 %; collectively, those QTL explained 37 % of the variance. For six out of the nine QTL, there was no significant interaction with environment detected at a genome-wide significance level of 5 %. These six QTL (on A2, A7, A8, C2, C4 and C8) were less sensitive to environmental factors and therefore considered to be stable across environments; especially, the QTL on A2 (detected in INRA95 and RRes09) and on A7 (detected in INRA95 and INRA07) were consistently detected in experiments over 15 years either in both France and the UK or within France (Fig. 2).

**Table 4:** QTL and QTL × E (environment) effects detected for resistance against *Leptosphaeria maculans* across five winter oilseed rape field experiments using the BnaDYDH (Darmor-*bzh* × Yudal) population

Linkage group^a^	Locus^b^	Position (cM)^b^	QTL main effect	% variance accounted for			QTL × E interaction?
				Main effect	Interaction	Total	
A2	H09.570, sR94102a	14.05	0.08	3.37	1.13	4.49	Yes
A2	E02.1200, ScJ14	94.10	0.14	6.43	0	6.43	No
A4	PFM628, sN2025	23.40	0.15	4.96	2.69	7.64	Yes
A6	Gai/Bzh	126.63	0.14	6.78	2.53	9.31	Yes
A7	A08.2340	39.10	0.10	1.56	0	1.56	No
A8	A08.960, IGF3222b	55.80	0.11	1.80	0	1.80	No
C2	Fad8	17.19	−0.16	1.83	0	1.83	No
C4	ScNP009, A09.1000	120.05	0.12	2.30	0	2.30	No
C8	CB10449	70.73	0.12	2.85	0	2.85	No

^a^LG, the linkage groups, are named according to *Brassica napus* A1–A10 and C1–C9 designations by the Multinational *Brassica* Genome Project Steering Committee

(http://www.brassica.info/information/lg_assigments.htm)

^b^The locus names indicate the markers nearest to the estimated QTL position. The loci are shown in Fig. 2

**Fig. 3:**
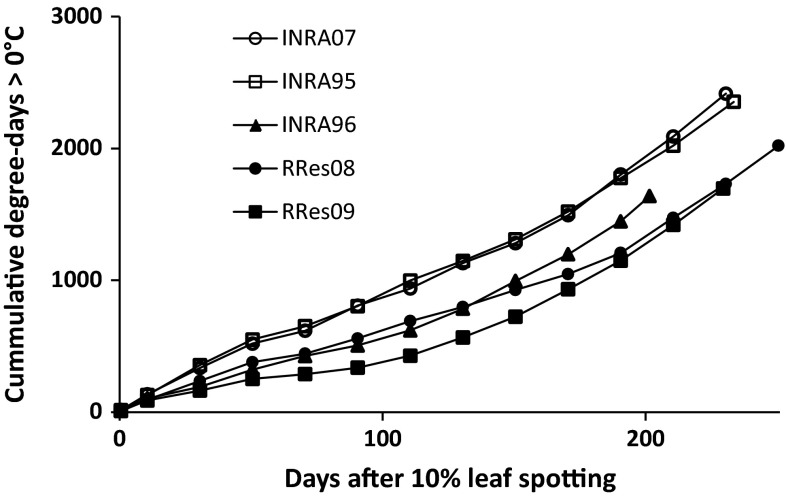
Cumulative degree-days from the date in autumn when 10 % of plants were observed with phoma leaf spots to the date before harvest in summer when phoma stem canker was assessed in five winter oilseed rape field experiments with the BnaDYDH (Darmor-*bzh* × Yudal) mapping population. For details about experiments, see Table 1

The temperature and rainfall associated with the five experiments differed between years and countries (S-Fig. 1). The severity of stem canker was influenced by the weather conditions, especially the temperature. In France, stem canker was more severe in INRA95 (1994/1995) and INRA07 (2006/2007) than in INRA96 (1995/1996) experiments; in the UK, stem canker was more severe in RRes09 (2008/2009) than in RRes08 (2007/2008) experiments (Table 1; Fig. 1). During the three cropping seasons in France, the monthly temperatures were generally greater in 1994/1995 and 2006/2007 than in 1995/1996; in the UK, the monthly temperatures were lower in 2008/2009 than in 2007/2008 from September to February (Table 5). In France, the mean temperature was greater in 1994/1995 (11.8 °C) and 2006/2007 (12.5 °C) cropping seasons than in 1995/96 (10.9 °C) season, while the mean daily rainfall was similar in the three cropping seasons (2.3 mm in 1994/95, 1.9 mm in 1995/1996 and 2.3 mm in 2006/2007). In the UK, the mean temperature in 2007/2008 (9.6 °C) cropping season was 0.5 °C greater than in 2008/2009 (9.1 °C) season, while the mean daily rainfall was similar in the two cropping seasons (2.0 mm in 2007/2008 and 2.1 mm in 2008/2009). In France, phoma leaf spots started earlier and the accumulated temperature (degree-days) from the date in autumn when 10 % of plants had phoma leaf spots increased more rapidly in 1994/1995 and 2006/2007 than in 1995/1996 (Fig. 3). The date when incidence of plants with phoma leaf spots first reached 10 % in 1995 (23 November 1995) was 27 or 30 days later than in 1994 (30 October 1994) and 2006 (24 October 2006). In the UK, although development of phoma leaf spots started later in 2008 (10 % plants with phoma leaf spots, 7 November 2008) than in 2007 (10 % plants with phoma leaf spots, 19 October 2007), the mean temperature during stem canker development stages (March to June) was greater; the more rapid increase in degree-days in spring led to more severe stem canker before harvest in the 2008/2009 season than in the 2007/2008 season. Comparing the five experiments, during the phoma leaf spotting period in autumn (September to December), the mean daily temperature and rainfall were, respectively, 2.9 °C and 0.5 mm greater in France than in the UK (Table 5). During phoma stem canker development period (March to June), the mean daily temperature was 1.5 °C greater in France than in the UK, while the mean daily rainfall was only 0.1 mm different between the two countries.

**Table 5:** Monthly mean temperature and mean rainfall during the growing seasons in five winter oilseed rape field experiments with the BnaDYDH (Darmor-*bzh* × Yudal) mapping population. For details about experiments, see Table 1

	Temperature (°C)					Rainfall (mm)				
	INRA95	INRA96	INRA07	RRes08	RRes09	INRA95	INRA96	INRA07	RRes08	RRes09
Aug	19.2	20.6	17.9	15.6	16.6	1.0	0.7	0.7	1.9	3.3
Sept	14.9	15.1	18.6	14.0	13.5	4.0	3.3	3.6	1.0	2.3
Oct	12.5	14.9	15.1	10.6	9.5	1.6	0.6	2.0	1.9	2.4
Nov	11.4	7.8	9.6	6.8	6.8	2.5	3.3	2.0	2.8	3.1
Dec	8.4	4.7	6.4	4.8	3.4	2.8	2.8	3.0	2.0	1.4
Jan	6.4	6.6	8.0	6.4	2.2	5.0	1.7	1.0	3.0	2.3
Feb	9.3	4.4	8.8	5.4	3.7	3.0	2.5	3.1	0.7	2.6
Mar	7.7	7.3	8.1	5.9	6.9	1.9	1.7	1.8	2.9	1.2
Apr	10.3	10.3	13.8	8.0	10.0	1.0	1.5	1.2	1.7	1.6
May	13.7	11.3	14.6	13.4	12.4	1.8	2.4	3.9	3.0	0.8
Jun	16.2	17.1	16.9	14.5	15.0	0.4	0.3	3.0	1.1	2.3
mean	11.8	10.9	12.5	9.6	9.1	2.3	1.9	2.3	2.0	2.1

## Discussion

These results suggest that it is possible to detect environmentally stable QTL for resistance against *L. maculans* in oilseed rape. Six stable QTL were identified by analysing the interactions between QTL and environment over five field experiments in France and the UK. Four of these QTL, on linkage groups A2, C2, C4 and C8, had previously been identified as stable QTL across two mapping populations (Pilet et al. 2001). The consistency of these results with those of the previous work suggests that these four QTL will be valuable for breeding cultivars with quantitative resistance against *L. maculans*. A knowledge about the stability of these QTL across different environments and different genetic backgrounds will increase confidence in using them in programmes for breeding resistance against *L. maculans* with marker-assisted selection.

Genetic analysis showed that quantitative resistance against *L. maculans* had a high heritability, which was consistent with previous results (Pilet et al. 1998). This high heritability (ranging from 0.82 to 0.94) across the five field experiments, together with the stability of many QTL, suggest that these QTL can be effectively used to breed for improved resistance against *L. maculans*. Among the six stable QTL, one on A2 may be particularly valuable because it had a greater effect than the other five QTL and showed no interaction with environment. Furthermore, the effect of this QTL was validated in the field experiments with near isogenic lines that differed in alleles for the corresponding chromosomal region in four different locations in France (Delourme et al. 2008b). This confirms the consistency of this QTL effect.

In this study, use of a new genetic map (Jestin et al. 2012) enabled detection of new QTL for resistance to *L. maculans* by re-analysis of data from INRA95 and INRA96. These QTL had not been detected using the previous map (Pilet et al. 1998), while most of the QTL detected previously were also detected in the new analysis. Since the genome coverage level of the genetic map affects the sensitivity of QTL detection (Asíns 2002), the differences in numbers of QTL detected probably resulted from differences in marker densities. In the work of Pilet et al. (1998), the genetic map contained 288 markers. With the development of new markers (Delourme et al. 2008b; Jestin et al. 2012) and publication of new marker resources (Wang et al. 2011a), the new map contained 505 markers. Most of the QTL detected in the 2006/2007 field experiment in France had been previously identified in 1994/1995 and 1995/1996, but two new QTL were identified. One of the new QTL on A1 had a large effect; the other one on A3 had small effect. One QTL on A6 with a large effect was identified close to the dwarf gene (*bzh*) in 1995/1996 in France and in 2008/2009 in the UK, when the stem canker severity was less. This suggests that the dwarf gene may affect the expression of resistance and detection of QTL in cropping seasons when the phoma stem canker is not severe (Pilet et al. 1998).

Fewer QTL were detected in the UK field experiments than in the French field experiments. All QTL detected in the UK were also detected in France, but one QTL on C1 with a strong effect detected in 2007/2008 in the UK had not been identified previously (Table 3, S-Table 4). These differences between different years and different locations might have occurred because the size of the mapping population used in the UK was smaller than that used in France or/and the stem canker severity in the UK was less than that in France; they might also have been caused by differences in pathogen populations. The accuracy of QTL detection has been shown to be very dependent on the population size (Charcosset and Gallais 1996). Differences in the size of the population could explain the differences observed between INRA07 and INRA95 or INRA96 experiments as well as differences between French and UK experiments. However, results of this study suggest that environmental factors, especially temperature, might also affect the severity of phoma stem canker and subsequent detection of QTL. For the three cropping seasons in France, the greater mean temperature in 1994/1995 and 2006/2007 cropping seasons than in 1995/1996 season was associated with more severe stem canker. Consequently, more QTL were detected in 1994/1995 and 2006/2007 than in 1995/1996, while the mean rainfall in the three cropping seasons was similar. The mean monthly temperatures of the three cropping seasons in France were 1–4 °C greater than those in the UK (Table 5). The greater temperature in France than in the UK, especially during the phoma leaf spotting period in autumn (September to December) and phoma stem canker development period in spring/summer (March to June), led to more severe stem canker in France than in the UK. Previous studies showed that environmental factors, especially temperature, affect both *R* gene resistance and quantitative resistance against *L. maculans* (Huang et al. 2006, 2009). The differences between the UK and France in disease severity and QTL detection might have also been influenced by the genetic composition of the *L. maculans* populations. Since the BnaDYDH population does not segregate for the major resistance genes that are effective or partly effective in the UK or France, the difference in phoma stem canker severity might have resulted from differences in aggressiveness between the *L. maculans* populations. However, no information on variation in aggressiveness of *L. maculans* isolates collected from the UK and France is available.

Our study clearly shows the need for multi-year and multi-location experiments and the usefulness of a combined analysis testing genotype × environment interactions in order to detect stable QTL. The detection of stable QTL for resistance against *L. maculans* will help with the fine mapping of the QTL. Recently, two *R* genes (*LepR3* and *Rlm2*) against *L. maculans* have been cloned (Larkan et al. 2013, 2015), and it has been proposed that such *R* genes operating against extracellular pathogens code for receptor like proteins (Stotz et al. 2014). However, no QTL against *L. maculans* have been cloned. By comparison with *R* gene-mediated resistance, mechanisms of operation of quantitative resistance are poorly understood (Poland et al. 2008). With the available genome sequences of the *Brassica* host (Chalhoub et al. 2014; Hayward et al. 2012; Wang et al. 2011b) and high marker density genetic maps (Delourme et al. 2013; Wang et al. 2011a), it is now possible to perform genome-wide association mapping (Fopa Fomeju et al. 2014) and to develop markers linked to the stable QTL. These markers will be valuable for marker-assisted breeding for durable resistance against *L. maculans,* a damaging disease of an economically important crop, which will contribute to food security.

### Author contribution statement

Conceived and designed the experiments: YJH, RD, GJK, BDLF, MJM; performed the experiments: YJH, CJ; analysed the data: YJH, SJW, RD, CJ; wrote the paper: YJH, BDLF, RD, SJW, MJM, GJK.

## Electronic supplementary material

Supplementary material 1 (DOCX 127 kb)

Supplementary material 2 (DOCX 26 kb)

Supplementary material 3 (XLSX 14 kb)

Supplementary material 4 (XLSX 31 kb)
