# Supplementary figures and images for: Identification of environmentally stable QTL for resistance against Leptosphaeria maculans in oilseed rape (Brassica napus)
Source: Theor Appl Genet. 2015 Oct 30;129:169–80. doi: 10.1007/s00122-015-2620-z (PMC4703627; doi:10.1007/s00122-015-2620-z)

**Supplementary Figure 1**


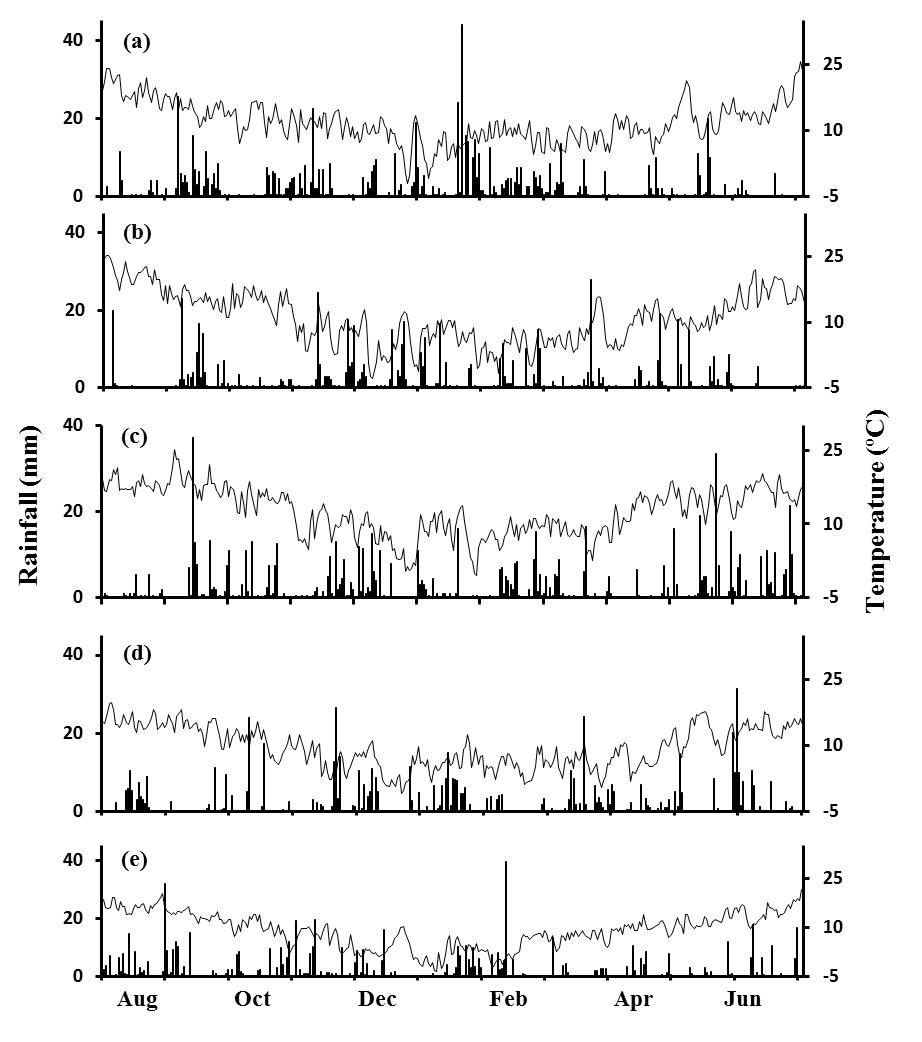

Supplement: Supplementary file 1 — Supplementary material 1 (DOCX 127 kb) [file 122_2015_2620_MOESM1_ESM.docx]
