# Supplementary material for: Identification of environmentally stable QTL for resistance against Leptosphaeria maculans in oilseed rape (Brassica napus)
Source: Theor Appl Genet. 2015 Oct 30;129:169–80. doi: 10.1007/s00122-015-2620-z (PMC4703627; doi:10.1007/s00122-015-2620-z)
Supplement: Supplementary file 2 — Supplementary material 2 (DOCX 26 kb) [file 122_2015_2620_MOESM2_ESM.docx]

**Supplementary tables**

**S-Table 1** Phoma stem canker severity (G2 index) assessed before harvest of the BnaDYDH mapping population parental lines and the control cultivars in five winter oilseed rape field experiments

|  | Phoma stem canker severity (G2 index) | | | | |
| --- | --- | --- | --- | --- | --- |
|  | INRA95 ^a^ | INRA96 | INRA07 | RRes08 | RRes09 |
| Parental lines |  |  |  |  |  |
| Darmor-*bzh* | 3.17 | 1.98 | 2.19 | 0.54 | 1.13 |
| Yudal | 6.72 | 7.47 | 7.77 | 4.17 | 7.00 |
|  |  |  |  |  |  |
| Control cultivars | | | | | |
| Aviso | - ^b^ | - | 2.34 | 0.58 | - |
| Canberra | - | - | - | 0.65 | - |
| Darmor | - | - | 2.39 | 0.46 | 1.22 |
| Eurol | 5.93 | 5.38 | 4.59 | 1.60 | 3.80 |
| Falcon | 4.39 | 3.90 | 3.35 | - | - |
| Jet Neuf | - | - | 1.77 | - | - |
| NK-Bravour | - | - | - | - | 1.72 |

^a^ Details of field experiments are given in Table 1

^b^ Not tested

S-Table 2 and S-Table 3 are too big, so they are presented as Excel files.

**S-Table 2** Genetic map used for QTL detection

**S-Table 3** Primer information for the marker loci located at the peak or flanking the peak of the QTL

**S-Table 4** Summary of QTL for resistance against *Leptosphaeria maculans* detected by composite interval mapping in previous studies (Pilet et al., 1998; Jestin et al., 2012) and this study using three winter oilseed rape field experiments (INRA95, INRA96, INRA07) with the BnaDYDH (Darmor-*bzh* × Yudal) mapping population

| Current study | | | Previous study | | |
| --- | --- | --- | --- | --- | --- |
| LG^a^ | Locus ^b^ | Position (cM) | LG ^c^ | Locus ^b^ | Position (cM) |
| INRA95 |  |  |  |  |  |
| A2 | E02.1200 | 89.7 | A2– DY11 | K08.2130 | - ^d^ |
| A4 | CB10347 | 4.2 |  |  |  |
| A6 | A18.1580 | 88.5 | A6 – DY6 | W05.750 | - |
| A7 | CB10450 | 31.3 | A7–DY10 | U01.690 | - |
| A8 | CB10013b | 43.1 | A8 – DY9 | O13.490 | - |
| A9 | W15.1470 | 124.8 | A9 – DY5 | W11.1890 | - |
| C2 | Fad8 | 17.2 | C2 – DY2 | L08.1190 | - |
| C4 | A09.1000 | 120.0 | C4 – DY3 | P05.1375 | - |
| INRA96 |  |  |  |  |  |
| A1 |  |  | A1– DY1 | C02.860 | - |
| A6 | Bzh | 130.0 | A6 – DY6 | M07.730 | - |
| C2 | W11.610 | 52.5 | C2 – DY2 | L08.1190 | - |
| C4 |  |  | C4 – DY3 | P05.1375 | - |
| C7 | Na12A10 | 123.1 |  |  |  |
| C8 | H06.CD1 | 102.7 | C8 – DY8 | E11.2150 | - |
| INRA2007 |  |  |  |  |  |
| A1 | sN2305a | 104.9 | A1 | sN2305a | 114.7 |
| A2 | sR94102a | 5.0 | A2 | sR94102a | 11.3 |
| A2 |  |  | A2 | ScJ14 | 103.5 |
| A3 | Na12007 | 215.6 | A3 | Na12C07 | 230.5 |
| A4 | sN2025 | 28.1 | A4 | CB10448 | 2.0 |
| A6 | PFM191a | 69.0 |  |  |  |
| A7 | A08.2340 | 39.1 | A7 | A08.2340 | 44.4 |
| A9 | ScL12 | 105.9 | A9 | CZ0b697380 | 68.9 |
| C4 |  |  | C4 | CB10107 | 60.0 |
| C7 | Bras014 | 83.5 | C7 | Bras014 | 94.1 |
| C8 | CB10449 | 70.7 | C8 | H06.CD1 | 119.5 |

^a^LG, the linkage groups are named according to *Brassica napus* A1–A10 and C1–C9 designations by the Multinational *Brassica* Genome Project Steering Committee

(<http://www.brassica.info/information/lg_assigments.htm>)

^b^The locus names indicate the markers nearest to the estimated QTL position

^c^LG, the linkage groups were previously names as ‘DY’ in corresponding to *Brassica napus* A1–A10 and C1–C9 designations

^d^ The map position was not indicated for INRA95 and INRA96 data because the map used was much less complete than the ones used in Jestin et al (2012) or in this study.

**S- Fig. 1** The daily temperature (°C) and rainfall (mm) in France and the UK at the sites of five winter oilseed rape field experiments during five growing seasons: a, INRA-Rennes, France 1994/1995; b, INRA-Rennes, France, 1995/1996; c, INRA-Rennes, France, 2006/2007; d, Rothamsted, UK, 2007/2008; e, Rothamsted, UK, 2008/2009. Further details of these experiments are given in Table 1.
